# 728. Genomic Characterization of *Burkholderia pseudomallei* Isolates in Colombia

**DOI:** 10.1093/ofid/ofab466.925

**Published:** 2021-12-04

**Authors:** Catalina Espitia-Acero, Rafael Rios, Monica Gabriela huertas, Sandra Vargas, Carolina Duarte Valderrama, Jaime Moreno Castañeda, Jose Y Rodriguez, Carlos A Alvarez-Moreno, Nataly Camargo, Giovana Katherinne Casadiego-Santiago, Soraya Salcedo, Sandra Rincon, Cesar A Arias, Lorena Diaz, Lorena Diaz, Jinnethe Reyes

**Affiliations:** 1 Universidad el Bosque, Bogota, Distrito Capital de Bogota, Colombia; 2 Universidad El Bosque, Bogota, Distrito Capital de Bogota, Colombia; 3 Instituto Nacional de Salud, Bogotá, Distrito Capital de Bogota, Colombia; 4 Centro de Investigaciones Microbiológicas del Cesar, Valledupar, Cesar, Colombia; 5 Clínica Colsanitas, Clínica Universitaria Colombia, Bogotá, Distrito Capital de Bogota, Colombia; 6 Clinica General del Norte, Barranquilla, Atlantico, Colombia; 7 Clínica General del Norte. Universidad Simón Bolívar, Barranquilla, Atlantico, Colombia; 8 CARMiG, UTHealth and Center for Infectious Diseases, UTHealth School of Public Health, Houston, TX; Molecular Genetics and Antimicrobial Resistance Unit and International Center for Microbial Genomics, Universidad El Bosque, BOG, COL, Houston, TX; 9 Molecular Genetics and Antimicrobial Resistance Unit and International Center for Microbial Genomics, Universidad El Bosque, Bogota, Distrito Capital de Bogota, Colombia

## Abstract

**Background:**

Melioidosis is a serious infection caused by *Burkholderia pseudomallei* (Bps), an opportunistic organism, highly adaptable and with a wide array of intrinsic virulence factors and antimicrobial resistance determinants. Bps is underdiagnosed due to its slow growth on routine laboratory media and the lack of robust diagnostic infrastructure in rural areas of low/middle income countries. Recent data indicates that Bps infections are increasing in Colombia (COL). However, the understanding of the genomic epidemiology and population structure of the emerging Bps isolates in COL is unknown. Here we characterize the genomic features of Bps isolates from infected patients in COL.

**Methods:**

We identified 13 Bps clinical isolates recovered in 5 Colombian cities between 2018 and 2020. We performed WGS and phylogenomic analyses using Bayesian methods. For comparisons, we included 82 publicly available genomes from Bps recovered worldwide (including 10 additional isolates from COL). Additionally, we characterized the resistome, virulome and MLST of all isolates.

**Results:**

12 out of the 13 isolates were confirmed as Bps and 1 belonged to the *B. cepacia* complex. The Bps population structure was divided in two main clades: clade 1 with isolates from Asia and Australia, and clade 2 with isolates from Africa, America, and the Caribbean (Figure 1). We found two groups of Colombian isolates, the first was related to ST518 and the second, highly diverse including 11 different STs (1742, 1748, 92, among others). Genomic characterization showed the presence of β-lactamases PenA (n=11) and OXA-57 (n=1). We also identified a T584A substitution in PBP3 (n=11). All genomes contained virulence determinants of motility (BimA), invasion (Flagella), signaling (CdpA) and adherence (Type IV pili). Type III and VI secretion systems, were also found in all isolates resembling Bps from other parts of the world.

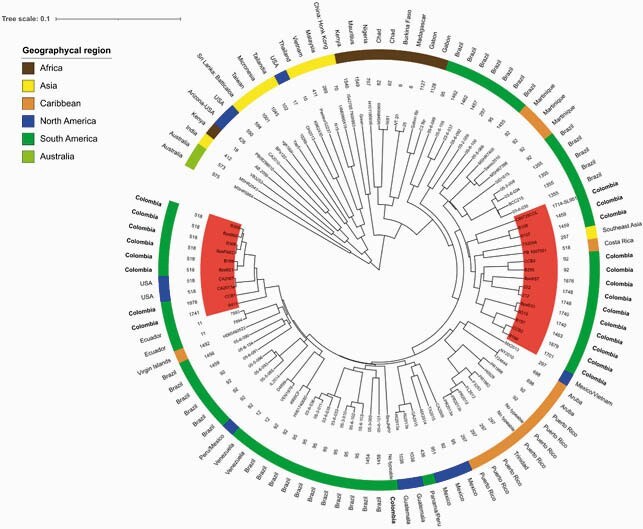

Figure 1. Maximum clade credibility tree of 82 genomes of Bps. The inner ring shows the ST for each genome, while the outer ring shows the geographical region associated with them. Groups highlighted in red show the location of the Colombian genomes and those related to them.

**Conclusion:**

Bps is an emerging pathogen in COL and its population structure seems highly diverse, predominantly of the American lineage and absence of Australasians strains. A high prevalence ( >90%) of resistance determinants, particularly related to β-lactams, suggest that active surveillance of these emergent pathogens is needed in countries like COL.

**Disclosures:**

**Cesar A. Arias, M.D., MSc, Ph.D., FIDSA**, **Entasis Therapeutics** (Grant/Research Support)**MeMed Diagnostics** (Grant/Research Support)**Merk** (Grant/Research Support) **Lorena Diaz, PhD** , Nothing to disclose

